# Epigenetic Links between Microbiota and Gestational Diabetes

**DOI:** 10.3390/ijms23031831

**Published:** 2022-02-06

**Authors:** Olimpia Mora-Janiszewska, Anna Faryniak-Zuzak, Dorota Darmochwał-Kolarz

**Affiliations:** Department of Obstetrics and Gynaecology, Medical College, University of Rzeszów, 35-959 Rzeszów, Poland; olimpiajaniszewska@gmail.com (O.M.-J.); ddarmochwal@ur.edu.pl (D.D.-K.)

**Keywords:** gestational diabetes mellitus, epigenetics, microbiota

## Abstract

Gestational diabetes mellitus (GDM) is considered a significant and increasing worldwide problem. The growing body of evidence on this topic has allowed us to point out that a hostile intrauterine environment in mothers with GDM via epigenetic mechanisms induces “diabetogenic” and “obesogenic” changes in an offspring’s DNA. This sets a vicious intergenerational cycle of metabolic diseases in motion, gradually deteriorating the health of the human population. One of the most important participants of this process seems to be altered microbiota. There is a chance that the identification of specific epigenetic marks may provide a key for future diagnostic, prognostic and therapeutic solutions in the field of personalised medicine. Given the reversibility of most epigenetic changes, there is an opportunity to improve the long-term health of the human population. In this manuscript, we aim to summarise available data on epigenetic changes among women suffering from GDM and their progeny, in association with alterations in the microbiome.

## 1. Introduction

Diabetes mellitus is a global metabolic disease. A significant increase in its incidence rate has been observed for several years. According to data provided by WHO, the total number of people with diabetes has quadrupled in the past 40 years. It is estimated that 425 million people worldwide have diabetes, however, the number of undetected cases remains unknown. In 2025, it is projected that 570 million people will be diabetic, causing 1.6 million deaths [1]. A continuously increasing prevalence of gestational diabetes is also observed, thus, GDM still remains one of the most common complications of pregnancy [2,3,4]. According to the American Diabetes Association (ADA), GDM is defined as diabetes recognised in the second or third trimester, provided that overt diabetes had been excluded priorly, or, at the latest, in early pregnancy [5].

GDM usually resolves after delivery, but unfortunately, it may induce long-lasting metabolic and cardiovascular complications, such as type II diabetes mellitus (T2DM) and cardiovascular disease in the mother, enhanced adiposity or even obesity, impaired glucose metabolism, hypertension, hyperlipidaemia, non-alcoholic fatty liver disease in the offspring, as well as pre-term puberty [6].

So far, many risk factors of gestational diabetes have been identified and widely described. Being overweight during pregnancy or maternal obesity, increased weight gain during the first or early second trimester, advanced reproductive age, family history of type 2 or gestational diabetes, foetal macrosomia in prior pregnancy and polycystic ovary syndrome are considered significant factors predisposing to this disease [7,8,9,10,11,12,13,14,15].

Although numerous risk factors have been identified and screening in high-risk groups has been introduced, the upward trend in the prevalence of gestational diabetes and its complications still remain. Thus, there is an urgent need to identify sensitive and specific biomarkers relevant for the early detection of GDM.

The human gut microbiota consists of approximately 1000 trillion microbes: bacteria, viruses, archaea and eukariota [16]. It is estimated that the absolute mass of the microbiome in the human body reaches approximately 2 kilogrammes [17]. The microbiome composition seems to have a significant impact on metabolic processes and, therefore, on human health [18]. Pathologically altered gut microbiota seem to contribute to the development of diabetes GDM in the mother, and, consequently, may increase the risk of “diabetogenic” and “obesogenic” changes in her own DNA and in that of the offspring through epigenetic alterations.

Epigenetic modifications are defined as changes in gene expression without nucleotide sequence alterations. It is believed that the essential function of epigenetics in the genome surroundings is to provide a response to internal and external environmental factors through dynamic, mostly reversible, changes in chromatin structure and gene expression. The basic epigenetic processes are considered to be DNA methylation, histone modifications, noncoding RNA regulation and chromatin remodelling. 

In our review, we attempted to emphasize that gut microbiota dysbiosis, occurring via epigenetic pathways, may contribute to metabolic complications, not only in affected mothers, but also in their offspring. In this manner, pathologic changes can be transferred to subsequent generations, increasing the frequency of obesity, diabetes and inflammatory diseases, which concerns even younger individuals. Given that most epigenetic changes are reversible, the identified epigenetic marks may become important diagnostic, prognostic and therapeutic targets.

## 2. Pathogenesis and Pathophysiology of Gestational Diabetes

The pathophysiology of GDM is not fully understood, with the prevailing hypothesis linking abnormal placental hormone expression to maternal metabolic dysfunction and, in particular, the synthesis and function of insulin. During physiological pregnancy, enhanced production and secretion of insulin, which is possible due to the hypertrophy and proliferation of pancreatic beta cells [19,20], is accompanied by an alteration in the sensitivity of tissues to insulin. In the early stage of gestation, insulin sensitivity starts to increase from the 14th week, however, gradually rising insulin resistance is observed, reaching its peak by late pregnancy [21]. The insulin resistance phenomenon is believed to be associated with enhanced concentrations of local and placental hormones such as placental lactogen, placental growth hormone, oestrogens, progesterone, prolactin, adiponectin and leptin [22,23,24]. The pro-inflammatory state present in every normal pregnancy is also considered as an essential contributing factor. Increased concentrations of prodiabetogenic hormones and pro-inflammatory changes at a cellular level contribute to an overall increase in insulin resistance, which is aimed at ensuring an adequate supply of glucose to the foetus [25,26,27,28,29].

In the course of GDM, however, an increased concentration of pro-inflammatory markers, such as CRP, TNF-alpha, IL-6, disturbed adaptation of pancreatic beta cells to altered metabolic conditions and impairment of the insulin signalling pathway are observed. The insufficient compensatory increase in insulin secretion is noted, which leads to hyperglycaemia under the conditions of a greater decrease in insulin sensitivity than in normal pregnancy. Foetal hyperinsulinemia, due to maternal hyperglycaemia, drives metabolic disturbances in the foetus [30,31,32,33].

## 3. The Role of Microbiota in GDM Development

Microbiota are found in specific places such as the mouth, skin, digestive system, upper respiratory tract and the reproductive system, but the most numerous and active is the gut microbiome. Firmicutes and Bacteroidetes account for 80–90% of the intestinal bacterial microbiome; less numerous are Proteobacteria, Verrucomicrobia, Actinobacteria and Fusobacteria. The human gut microbiome composition alters from early life to older age. In order to ensure homeostasis, the microbiota constantly adjust and vigorously react to a variety of external and internal stimuli [34,35].

The growing interest in the influence of the microbiome on human health has resulted in the commencement of the Human Microbiome Project, carried out by the National Institute of Health in 2008. The project was aimed at detecting differences in the human microbiome, depending on the studied population, their genotype, health state, age, nutrition, applied treatment, living environment and social factors [17]. The methods for studying the human microbiome were based on an analysis of the 16S rRNA sequence and sequencing of the metagenome [36].

During pregnancy, changes in the composition and functioning of the microbiome occur. The gut microbiota alters during each trimester of pregnancy. The number of bacteria in the intestinal microbiota increases, while its composition changes [37]. To allow normal foetal growth, an incremental shift is observed towards microorganisms involved in energy production and accumulation. The increase in Akkermansia, Bifidobacterium and Firmicutes appears parallel to the rising energy storage, while the increase in Proteobacteria and Actinobacteria helps protect the foeto-maternal unit from external infection [35]. Along with the increase in gestational age, Firmicutes begin to dominate, similarly as in the case of microbiota in obese people [38,39]. The number of Faecalibacterium taxomes and other producers of short-chain fatty acids decreases [40], causing reduced production of butyric acid with proven anti-inflammatory properties [41]. Both in normal pregnancy and that complicated with GDM, the abundance of Blautia and Collinsella increases [42]. In GDM, the Firmicute/Bacteroides ratio increases compared to healthy pregnant women [43]. Crusell et al. presented Actinobacteria, Collinsella, Rothia, Actinomyces, Desulfovibrio, Leuconostoc, Granulicatella and Mogibacterium as GDM biomarkers, while Marvinbryantia, Acetivibrio and Anaerosporobacter were exhibited as markers of normal carbohydrate metabolism in pregnancy [39]. In another study, Bacteroides, Dialister and Campylobacter were indicated as biomarkers of GDM, while Gemminer and Bifidobacterium were noted as markers of normal blood glucose levels during pregnancy [44]. In women with GDM, in the third trimester of pregnancy, increases in the number of Bacteroides caccae, Bacteroides massiliensis and Bacteroides thetaiotaomicron, as well as a reduction of Bacteroides vulgatus, Eubacterium eligens, Lactobacillus rogosae and Prevotella copri, have been observed [45]. The relationship between an increase in the relative abundance of Ruminococcaceae in early pregnancy and the later development of GDM [46,47] is also known. Moreover, the maintenance of a changed composition of the microbiota after childbirth was observed [39]. A higher glucose level in pregnant women is positively correlated with the wider range of taxomes in gut microbiota [47]. Reports from recent research have allowed us to identify various microorganisms within the intestinal microbiota of women with GDM. Ordinarily, in these studies, the composition of the microbiota among a control group is compared with women at a high risk of or diagnosed with GDM. An overview of the research results from recent years is presented in Table 1.

Interesting findings were noted by Hu et al. in their study based on measuring the quality of microbiota in women, comparing the first trimester (6–15 hbd) with the late second (24–28 hbd). The study was conducted on a numerically equivalent group of women with GDM and controls. Strong dependence on the prevalence regarding the dominance of Enterobacteriaceae, *Ruminococcaceae* spp. and *Veillonellaceae* spp. in the group of women with GDM has been demonstrated [51].

In a study by Ma et al., the microbiota of women with GDM in the first trimester of pregnancy (10–14 hbd) and after the post-partum period was compared in 2 equal groups. The Eisenbergiella, Tyzzerella and Lachnospiraceae NK4A136 species were also highly prevalent 42 days after delivery in women with GDM [53].

The available studies also allowed us to note a depletion in the presence of some bacterial taxomes concerning the microbiota of women with GDM, compared to pregnant women with normoglycaemia. This concerned the Faecalibacterium [53], *Bifidobacterium* spp., *Eubacterium* spp. [54], *Marvinbryantia*, *Acetivibrio*, *Anaerosporobacter* [39] and *Bacteroides* spp. groups [43]. These taxa can be potential predictors of normoglycaemia.

The observations discussed above are summarised in Table 2.

In the literature, there is a constantly growing body of evidence regarding the influence of microbiota on the development of metabolic syndrome, obesity and diabetes mellitus [38,39,55,56]. It is believed that microbial metabolites play a key role in the microbiota-host axis [57]. The influence of microbial short-chain fatty acids, lipopolysaccharide and trimethylamine on the development of obesity and metabolic syndrome is known [57,58,59]. Physiological dysbiosis occurring in the third trimester of pregnancy has a direct, positive influence on the formation of low-inflammatory processes, hyperglycaemia, excessive weight gain and insulin resistance [1,60,61].

In pregnant women, the phenomenon of higher numbers regarding Gram-negative bacteria and the disturbance of the Gram-negative/-positive bacteria ratio is well-known. Lipopolysaccharides (LPS) constitute a large proportion of the Gram-negative bacteria cell wall. The increase in the level of LPS in the intestinal microflora contributes to the induction of metabolic endotoxemia and the production of low-grade inflammation [62]. In animal models, an increase in the activity of Toll-like receptors (TLRs) and subsequent mobilisation of inflammatory vectors has been demonstrated. Several pro-inflammatory pathways have been identified, which are mediated by the interleukin receptor associated kinase (IRAK), TGF-1 related kinase, NFkB, IKK-β, INK., etc. This leads to the development of low-grade inflammation, infiltration of macrophages and an increase in the concentration of pro-inflammatory cytokines, including IL-1β, IL-6 and TNF-α [62,63,64,65,66]. Pro-inflammatory cytokines induce a state of insulin resistance in nephralgic locations—the liver, muscles and adipose tissue. In adipocytes, phosphorylation of serine residues occurs at the level of insulin receptor substrate proteins (IRS) by the kinase activated via the mutation of the p38 gene (MAPK). In microtubules, the process of phosphorylation takes place at the serine residue—307 (Ser307) by MAPK and kB inhibitor kinase [67]. Interleukin 1β significantly influences the failure of beta cells in pancreatic islets. During glucose stimulation, insulin production in beta cells is intensified and inflammatory processes dependent on interleukin 1β and interleukin receptors (IL-1R) are intensified. These processes lead to the dysfunction and apoptosis of pancreatic islet beta cells [67,68]. In recent years, clinical trials have been carried out on the use of IL-1 receptor antagonists (IL-1R) in the treatment of diabetes. Test substances include canakinumab and gevokizumab, known as XOMA 052 [69,70,71]. For unarguable reasons, there are no studies on the use of IL-1R antagonists in pregnant women.

Koren et al. analysed stool probes from 91 pregnant women, previously recruited for a prospective, randomised, mother–infant pair-study in Finland. They described a significant alteration in the gut microbiota between the first and third trimesters. Although they did not find any differences between the microbiota of the pregnant women in the first trimester or that compared to their normal healthy controls, the differences were significant in the third trimester. The between-subject diversity has greatly expanded, and enrichment of Proteobacteria and Actinobacteria has been observed in the majority of pregnant women. In the third trimester, in most women, an increase in Proteobacteria level has been previously reported in the case of inflammation-related dysbiosis. *Faecalibacterium*, on the other hand, being an anti-inflammatory butyrate producer, was, on average, less abundant in late pregnancy [72]. They also transferred gut microbiota from the first and third trimester in women to germ-free recipient mice and noticed that the latter induced greater adiposity insulin resistance, in addition to inflammatory response. Increased concentration of pro-inflammatory cytokines, such as: IL-1β, IL-2, IL-5, IL-6 and GM-CSF, was observed [72].

The “leaky gut phenomenon” is a widely studied hypothesis that can explain the mechanisms of pathobiom invasion into the mesentery and blood vessels. In addition to phagocytosis and receptor-dependent active mechanisms, mechanical potential is also shown, which is dependent on defects in the mucosa and tight junction proteins. There is ample evidence suggesting the involvement of *Prevotella* spp. in the degradation of the mucin covering the intestinal villi cells [73,74]. In the work by Cani et al. and Bagarolli et al., it was also proved that the change in intestinal microflora negatively influences the expression of adherent proteins ZO-1 and occludin, increasing the mechanical ability of the pathobiom leak [75,76]. In these studies, the influence of a high-fat diet on the negative regulation of intestinal tightness, depending on the mechanisms of the endocannabinoid system (eCB) [77,78,79], has been emphasised. In a study carried out by Bawah et al., a close relationship was described between the increase in plasma zonulin levels in pregnant women in the first trimester of pregnancy as a modulator of tight intercellular junctions and the risk of GDM [16,80].

It is suspected that microbiota metabolites play a significant part in maternal epigenetic regulation of GDM and in programming a child’s metabolite profile. In the publication by Zhao et al. [81], the relationship between maternal faecal metabolome and neonatal blood metabolome was investigated. The authors observed a negative prevalence in the presence of 4 different faecal metabolites in mothers with GDM compared to the control group: lysine, putrescine, guanidinoacetate and hexadecanedioate. These substances are known to play a protective or indicative role in the development of glucose metabolism disorders [53,82,83]. Zhao et al. also proved an increased level of biotin metabolism in patients with GDM. These observations may provide evidence of the significant contribution of maternal microbiota metabolites to inborn errors of metabolism (IEMs).

It seems likely that the metabolites of the gut microbiota may become recognised biomarkers of GDM in the future. The development of screening tests would allow for earlier identification of GDM and IEM risk and to take preventive measures [84].

In the literature on the subject, the hypothesis concerning the influence of internal (microbiota) and external (environment) factors on the regulation of gene expression within the context of the development of glucose metabolism disorders is currently being discussed. Still, there are a lack of publications on the direct influence of microbiota or its metabolites on specific genome sequences.

In the work by Kumar et al. [85], the effect of the Bacteroides, Firmicutes and Proteobacteria taxomes was investigated (present in the intestines of pregnant women) on DNA methylation of genes in inflammatory response, obesity and cardiovascular diseases. The presence of up- and down-regulation regarding many genes responsible for the development of metabolic disorders has been proven. Interestingly, several of these genes are also methylated in GDM patients, e.g., KCNIP3/4.

In a study conducted on whole genome research using the EWAS method, the relationship was examined between guanidine cytosine–phosphate–methylation and GDM. Nine sites of different CpG methylation were identified which, in the future, may be used as biomarkers in the early diagnosis of GDM patients [86].

A series of complex, biochemical, pro-inflammatory and microbiological mechanisms lead to insulin resistance and the development of GDM in pregnant women. There is an existing suggestion that, on the one hand, gut microbiota affect host metabolism but, on the other, the host can manage the gut microbiota to promote metabolic changes [80]. Although a relationship between the intestinal microflora and GDM has been proven, molecular mechanisms concerning the interaction of the intestinal microflora on the development of GDM are still largely unknown [87].

## 4. Overview of Epigenetics

Two major periods of epigenetic reprogramming (erasure and re-establishment) are known: in primary germ cells (PGCs) and from fertilisation to the pre-implantation stage. This is a period particularly vulnerable to the disruptive effects of internal and external factors. The then-created phenotypes and epimutations can be passed down through subsequent generations, thus making them significant underlying pathogenetic mechanisms for the development of complex multifactorial diseases. In this context, we refer to intergenerational epigenetic inheritance when direct exposure to a particular stressor cannot be excluded, and transgenerational inheritance of phenotypes and epimutations is maintained without any direct exposure to the stressor. In the maternal lineage, exposure during gestation implies direct exposure of the mother and the foetus (intergenerational), and their developing primary germ cells (transgenerational inheritance) [88].

The most frequently studied mechanism is the methylation of cytosine nitrogenous bases in the DNA chain. DNA methylation reactions are catalysed by DNA methyltransferases (DNMT). It is well-known that methylation is directly related to the inhibition of gene expression. The spots of increased methylation are CG dinucleotides within the cytosine bases, known as the CpG regions. It has also been proven that functionally active genes are hypomethylated, which enables the synthesis of proteins suitable for the metabolic tasks of a specified cell. Methylation, in the promoter region of a gene, reduces its expression, and methylation within the repressor of a given gene positively influences the expression of this particular gene. A number of consequences concerning disturbed cellular homeostasis have also been discovered, resulting in selective or global defects of genome methylation, including carcinogenesis processes [89,90].

Histone tail methylation may affect DNA methylation processes, and vice versa. The role of lysine amino acid trimethylation in histone H3 (H3U9, H3U27) and histone H4 (H4U20), as a condition of subsequent DNA methylation, has been demonstrated [91].

Another mechanism for modifying the chromatin strand and regulating gene expression is the modification of histones. The most widely described mechanisms are histone acetylation and deacetylation. These processes are catalysed by histone acetyltransferases (HAT) and histone deacetylases (HDACs), respectively [92].

The degree of chromatin folding is tightly regulated by the acetylation of lysine residues. Acetylation affects electrostatic relaxation between histones and phosphate residues. Therefore, this process leads to increased availability of the genome for the transcriptional apparatus and epigenetic mechanisms [93]. It has also been proved that the process of lysine deacetylation in histone 4 (H4U16) strongly influences folding of the chromatic thread [94].

Other mechanisms of epigenetic regulation include complex ATP-dependent processes of chromatin remodelling [95]. Gene expression is noted by modifying histones via non-coding RNA and miRNA [96].

Disturbances in mitotic and meiotic inheritance, as well as a simultaneous disturbance in the genetic balance of the cell, may lead to the development of multifactorial diseases, which include gastrointestinal neoplasms, arterial hypertension, as well as metabolic and haematological diseases. Recent years have also brought to light many novel insights into epigenetic mechanisms regarding the development of gestational diabetes [97].

## 5. The Role of Epigenetics in the Pathogenesis of GDM

An increasing number of studies on global methylation allow for more and more precise identification of specific methylation patterns in the course of several diseases, including DMG.

In pregnancy complicated by diabetes, DNA methylation is thought to be important in modulating the post-transcriptional profile of the genome. In studies conducted to date, functional panels of genes with altered methylation levels in placenta, cord blood or adipose tissue have been identified. For instance, in a cohort study conducted by Reichetreder et al., 1030 samples were tested for global methylation of placental DNA. A very strong correlation was found between maternal GDM and global DNA methylation (*p* = 0.0009). Variations related to clinical history, BMI, GDM predisposition and ethnicity were separated in this study. The authors suggest that GDM independently influences placental DNA methylation [98]. Ruchat et al. demonstrated differentially methylated genes (methylation difference eq. or more than 3%) in the placenta (3271 genes) and cord blood (3757 genes), including numerous genes associated with aberrant glucose metabolism. Interestingly, more than 25% of affected by GDM genes were common for placenta and cord blood, while 326 genes in placenta and 117 in the cord blood were also correlated with new-born weight [99]. Since the observed methylation changes occurred even in the presence of moderate hyperglycaemia, the authors concluded that even slight disturbances in the intrauterine environment may induce epigenetic changes that could influence the development of long-term complications such as obesity or type 2 diabetes in the offspring. In a recent study conducted by Chen et al., genome-wide DNA methylation was investigated in the peripheral blood, with regard to the relationship between intrauterine diabetes exposure, and the differentially methylated CpGs in 39 genomic regions identified in the offspring’s peripheral blood [38]. Methylation at three sites was also nominally associated with insulin secretion, while a fourth site was associated with future risk of T2DM [100]. Wu et al. showed significant differences in methylation patterns among women with GDM compared to healthy ones [101].

In a study by Ott et al., the authors demonstrated the variable nature regarding methylation of the IR transcription initiation regions within chromosome 19. A significant increase in methylation within region 1 and intron 1 was demonstrated. Moreover, the researchers demonstrated the highest level of DNA methylation in CpG in introns 1 in umbilical cord blood, then in maternal blood, visceral adipose and subcutaneous adipose tissue. Regarding region 1, methylation levels were relatively similar in all of the analysed tissues. In this study, the inverse relationship was demonstrated of methylation concerning the CpG4-GRE2, CpG3-AP-2 and SP1 regions with the expression of the IR gene in subcutaneous adipose tissue. The authors also showed that higher glucose levels were positively correlated with CpG2-AP-2 and SP1 methylation, while negatively associated with methylation of the CpG3-GRE1 region in umbilical cord blood cells of GDM-affected mothers [102].

Independently of studies on global DNA methylation in the placenta and cord blood, attempts are being made to associate methylation status of single genes coding proteins linked to GDM.

In the EPOCH study, Yang et al. [103] identified significant changes in 51 areas of the genome in samples of cord and peripheral blood from offspring of GDM and non-GDM mothers.

The methylation at differentially methylated positions in SH3PXD2A was significantly and positively correlated with adiposity-related parameters: BMI, waist circumference, triceps, suprailiac and subscapular skinfold thickness, subcutaneous adipose tissue quantity and leptin levels. DNA methylation in E2F6 was related to the fasting insulin and homeostatic model assessment for insulin resistance (HOMA-IR). According to researchers, these findings may suggest that DNA methylation is influenced by GDM exposure in the uterus and the subsequent epigenetic alterations may represent a significant link between this exposure and later metabolic consequences [104].

In the research by Haertle et al., the methylation level of many genes that may be associated with GDM was analysed. Significantly lower CpG2 region methylation levels of the gene encoding the alpha subunit of ATP- synthase F1 (ATP5F1A) in women with GDM were demonstrated. This study also allowed us to show the significant changes in methylation levels regarding the CpG4 regions for the MFAP4, CpG1, CpG2 and CpG3 gene in the PRKCH gene and in the CpG5, CpG6, CpG10 and CpG11 regions of the HIF3A gene in GDM. According to the authors’ opinion, the existence of epigenetic mechanisms at mitochondrial and extracellular levels in the epidemiology of GDM may be significantly suggested [105]. In the trial by Wu et al., 100 CpG islands, with significantly variable methylation, were identified, while the significant effect of the CpG variable methylation was detected for the genes of COPS8, PIK3R5, HAAO, CCDC124, C5orf34 proteins in GDM-affected women [106].

Kelstrup et al. showed lower expression of the PGC-1alpha (PPARGC1A) protein gene in the muscle tissue of the offspring of GDM mothers. The negative influence of PPARGC1A gene expression on HOMA-IR expression in subcutaneous adipose tissue has also been demonstrated. This suggests the influence of PPARGC1A expression on the development of insulin resistance and the influence of maternal overweightness on the onset of GDM [107]. These convergences were confirmed in research on the expression of PPAR gamma and PGC-1alpha carried out by Ruschle et al. [106].

In the study by Gague-Ouellet, focus was shifted towards the level of DNA methylation at the lipoprotein lipase (LPL) gene locus. LPL contributes to metabolic homeostasis through triglycerides hydrolysis and plays a pivotal role in lipid metabolism and transport. In peripheral vessels, as a result of hydrolysis, free fatty acids (FFAs) from circulating lipoproteins are released. The foetus is unable to synthetise enough FFAs, and thus depends on transplacental transfer.

The authors demonstrated a significant decrease regarding methylation in the CpG1 and CpG2 regions of the LPL gene in GDM-affected patients. A negative correlation was demonstrated between the methylation of the placental DNA concerning the LPL gene and its transcriptional activity. They also showed that alterations in foetal placental DNA methylation levels at the *LPL* gene locus that were associated with the anthropometric parameters in 5-year-old children. According to the authors, these findings support the concept of foetal metabolic programming through epigenetic changes [108].

The modification of histones in the genome also plays an important role in the mechanisms of epigenetic variation and seems to be an important factor in the pathophysiology of metabolic diseases and foetal programming, including GDM.

Hepp et al. investigated the level of lysine 9 acetylation and lysine 4 trimethylation within the H3histone (H3K9ac and H3K4me3, respectively) from placental tissue of 40 healthy and 40 GDM pregnancies. In this study, a significant reduction was demonstrated compared to the control group in H3K9ac expression concerning the syncytiotrophoblast, cells of the trophoblast, extracellular placental tissue and foetal endothelial cells in tissues derived from GDM-complicated placenta. No similar relationships were found in terms of H3K4me3 expression.

The researchers hypothesise that the down-regulation of H3K9c in the foetal endothelial cells may contribute to foetal programming of cardiovascular disease associated with GDM [109].

Chang et al. found that deacetylation of H3 and H4 histones, H3K4 demethylation and H3K9 methylation negatively affect the expression of PDX1 (IPF-1) [110]. IPF-1 deficits cause disorders in pancreatic beta cell maturation, leading to insulin secretion disorders [111]. In studies on animal models, the possibility has been proved of generation inheritance regarding disorders related to the dysfunction of PDX1 expression [112].

Another important mechanism of epigenetic regulation is the interaction of short, non-coding RNA, known as miRNA sequences. Zhao et al. found a protective role of miRNA-221 interacting with PAK1 towards pancreatic beta cells. In an animal model, the influence of miRNA-221 on insulin sequence, proliferation and inhibition of apoptosis concerning pancreatic islet cells was demonstrated. In the tested control group, significantly reduced expression of miRNA-221 was noted in individuals with GDM [113].

In the work by Stirm et al., a higher level of miRNA-340 expression was observed in the blood lymphocyte levels of mothers with GDM (*p* = 0.02) [114]. In other studies, an increased expression of miRNA-7-5p in women with GDM was also shown [115]. Moreover, research on animal models allowed us to demonstrate increased miRNA-143 expression and the possible generation-inheritance of glucose metabolism disorders [116].

The molecular mechanisms behind the effects of the microbiota on metabolism remain largely unknown, although recent evidence suggests that the gut microbiota plays a pivotal role in human metabolism and may be a significant factor affecting our epigenome. Among many important compounds synthesised by the gut microbiota, we may also find liposaccharides, folate, polyamines and enzymes, such as: methyltransferases, acetyltransferases, deacetylases, BirA ligases, phosphotransferases, which may act as epigenetic modulators taking part in DNA methylation and histone modification [117,118].

To date, unfortunately, only little research has been conducted among humans, focused on evaluating the correlations between microbiota and epigenetic modifications.

In a pilot study carried out among pregnant women, Kumar et al. investigated the association between gut microbiota and epigenetic changes and found a strong correlation between blood DNA methylation patterns and gut microbiota profiles. Clustering analysis of DNA methylome data revealed a clear correlation between the whole-blood epigenetic profile and composition of the gut microbial population among the mothers with a predominance of either Bacteroidetes and Proteobacteria (High Bact) or Firmicutes (High Firm). In mothers with higher Firmicute levels (High Firm), the authors found that promoters of 568 genes were more methylated, while the promoters of 245 genes were less methylated than in mothers with greater Bacteroidete and Proteobacteria levels. Among the affected genes, 82 are known to be associated with the risk of cardiovascular disease, 72 with lipid metabolism, 23 with obesity and 85 with inflammatory response. The most significant difference between the 2 groups was observed in methylation of the promoter region for SCD5, which was more highly methylated in the High Firm group and had undetectable methylation in the High Bact group (*p* = 0.00208). Lipopolysaccharide (LPS) was one of the up-stream regulators of genes identified in the network which, according to the authors, further strengthens the role of microbial molecules in epigenetic modifications [85].

In their work, Ramos-Molina et al. analysed DNA of gut microbiota composition in stool samples from 45 obese subjects by 16S ribosomal RNA (rRNA) gene sequencing. Twenty patients were selected on the basis of their Bacteroidete-to-Firmicute ratio (BFR). The authors found that in adipose tissue, both *HDAC7* and *IGF2BP2* were hypomethylated and over-expressed in the low BFR group compared to the high BFR one. They demonstrated that the DNA methylation status was correlated with gut microbiota composition in obese subjects and that the expression levels of candidate genes involved in glucose and energy homeostasis (e.g., *HDAC7* and *IGF2BP2*) could be epigenetically regulated by gut bacterial populations in adipose tissue. They further noted that hypomethylation in the *HDAC7* promoter, in both blood and fat tissue, is also related to impaired glucose metabolism, as distinct differences in glucose and HbA1c levels were observed in both study groups. This implies that alterations in the methylation profile of the HDAC7 gene are linked not only to the structure of the gut microbiota, but also to the metabolic status of the subjects, at least in the blood and adipose tissue [118].

In the study conducted by Tachibana et al., it was found that changes in the UBE2E2 and KCNQ1 methylation rates among umbilical cord samples were associated with the proportion of Firmicutes in the maternal gut, although with marginal correlations after adjustment for age and body mass index. These results may suggest a link between foetal diabetes-related gene methylation in foetuses and maternal microbiota components during pregnancy, but a limitation of this study is its small sample size [119]. 

In a randomised trial by Vähämiko et al., the authors aimed to assess whether probiotic supplementation throughout pregnancy may modify the DNA methylation status of gene promoters linked to obesity and weight gain in mothers and their children. They investigated the DNA methylation status of certain obesity promoters (623genes) and weight gain-related genes (433 genes) in mothers, as well as their offspring, and have concluded that probiotic supplementation resulted in markedly reduced DNA methylation levels in 37 gene promoters and increased DNA methylation levels in 1 gene promoter of women. In children, 68 gene promoters were significantly affected, with DNA methylation levels lower in the probiotic-treated group. They identified alterations in the epigenetic regulation of some components of insulin signalling pathways in response to the probiotic intervention, which may partly explain the favourable impact of probiotics on glucose metabolism. Remarkably, the promoter for insulin-like growth factor binding protein 1 (IGFBP1) was found to be less-methylated in both mothers and their children in the probiotic group. IGFBP1 encodes a protein that binds insulin-like growth factors I and II, and low levels of this protein have previously been linked to insulin resistance and diabetes [120].

Similarly, the MSRA (methionine sulfoxide reductase A) gene promoter was found to be less-methylated in the probiotic mother–children pairs. In an experiment on mice, animals lacking the MSRA gene showed reduced physiological insulin response in comparison to wild-type mice [33]. On this basis, the authors hypothesised that the reduced methylation of IGFBP1 and MSRA may be a possible mechanism for providing health benefits to both women and their children by diminishing the risk of impaired glucose metabolism. Researchers have suggested that probiotic supplementation during pregnancy, by targeting specific gene promoters, may provide beneficial long-term health effects. Nonetheless, a significant limitation of this study was the small size of the study group [120].

Further research is still required to fully understand the epigenetic mechanisms influencing GDM. Their results may be important in clinical practice.

In Table 3, the results obtained by the discussed research are presented.

## 6. Conclusions

The gut microbiota appear to play an important role in human metabolism and can be a significant environmental factor affecting our epigenome. In literature on the subject, the complex influence of epigenetic mechanisms is emphasized, including the effects of gut microbiota on the development of GDM in pregnant women. Currently, several dozen mechanisms of gene modulation at the mitochondrial and extracellular levels have been described. However, the authors of various studies highlight the lack of knowledge on the direct and indirect influence of epigenetic mechanisms on each other. An important question remains whether one regulation mechanism may affect the others in a cascade manner—the so-called “trigger effect”. The development of molecular techniques, e.g., RNA sequencing of the intestinal microbiome, may, in the future, allow us to find answers to many questions about the epigenetic mechanisms of genome regulation in pregnant patients. There is also no answer to the question regarding the risk-scale of intergenerational inheritance in terms of the effects of epigenetic mechanisms. A limitation of many available studies may be the failure to standardise patient groups in terms of BMI, race and origin, socioeconomic status and the number of offspring.

The currently available literature and the study findings cited therein point to a strong role of epigenetic mechanisms and the gut microbiota profile on the pathogenesis of GDM and human health programming in future ectopic life. The impact of epigenetic mechanisms on the pathophysiology of GDM and their long-term consequences for ectopic life require many years of wide-spectrum and complex research. The link between the gut microbiome and epigenome can be used as effective targets for the diagnosis and treatment of diseases. The composition of the gut microbiota may help us to understand the risk of developing GDM and therefore, increase the chance of detecting, preventing and treating this disease. There is an urgent need for further multi-directional, interventional and longitudinal studies on epigenetic modifications induced by microbiota alterations. Given that these studies are complex and costly, while awaiting their results, it seems valuable to attempt a broader introduction of recommendations that may serve to maintain eubiosis, especially among women of child-bearing age and, in particular, pregnant women. The periconceptional period, in addition to intrauterine development, is a unique time in the formation of the gut microbiota for the mother, but especially, for the foetus; therefore, they are essential for the programming of future human health. As epigenetic changes are mostly modifiable, there is a possibility to limit the intergenerational inheritance of metabolic traits and to reverse the unfavourable trend of the rising incidence regarding a broad spectrum of metabolic diseases and their serious long-term consequences. Undoubtedly, the reversibility of epigenetic changes should be taken into account, particularly within the context of GDM development. The conclusions of the currently available research might be incorporated into the protocols of perinatal counselling. After analysing the scientific evidence, it seems crucial to develop the aspect of social education in terms of GDM prevention, e.g., increasing awareness of transgenerational health, promoting the intake of well-balanced nutrition, especially the consumption of low glycaemic index foods, prebiotic products, and in high risk groups for GDM, the use of probiotics.

## Figures and Tables

**Table 1:** Recent studies showing the dominant taxonomies in the group of women with GDM.

Study	Number of Women Surveyed GDM/Control (Total)	Gestational Age (Weeks)	Methods	Prevailing Taxonomies in the Group of Women with GDM
**Su et al.** [48] nowe	21/32 (53)	24–28	16s rRNA sequencing	*Bacteroidetes*, *Bacteroides* spp., *Weisella* spp., *Fusicatenibacter* spp., *Parabacteroides*, *Roseburia*, *Flavonifractor*
**Sililas et al.** [49]	49/39 (88)	24–28	16s rRNA sequencing	*Bacteroidetes*, *Enterobacteriaceae*, *Clostridiales*, *Firmicutes/Bacteroidetes ratio*
**Wei et al.** [50]	15/18 (33)	24–28	16s rRNA sequencing	*Ruminococcus bromii*, *Clostridium colinum*, *Streptococcus infantis*
**Hu et al.** [51]	201/201 (402)	6–15 & 24–28	16s rRNA sequencing	*Enterobacteriaceae*, *Ruminococcaceae* spp., *Veillonellaceae*
**Chen et al.** [44]	30/28 (58)	28	16s rRNA microarray	*Corynebacterium* spp., *Lactobacillus* spp., *Blautia hydrogenotrophica*
**Chen et al.** [52]	110/220 (330)	25–26	16s rRNA sequencing	*Bacteroidetes* spp., *Dialister* spp., *Campylobacter* spp., *Enterococceae* spp.
**Ma et al.** [46]	70/70 (140)	10–14 & 42 days after delivery	16s rRNA sequencing	*Eisenbergiella*, *Tyzzerella*, *Lachnospiraceae NK4A136*
**Ye et al.** [53]	36/16 (52)	24–28	16s rRNA sequencing	*Blautia*, *Eubacterium halli*
**Cortez et al.** [43]	26/42 (68)	28–36	16s rRNA sequencing	*Ruminococcus*, *Prevotella*
**Crusell et al.** [39]	50/157 (207)	27–33	16s rRNA sequencing	*Desulfovibrio*, *Rothia* spp.
**Kuang et al.** [54]	43/81 (124)	21–29	Whole metagenome shotgun sequencing	*Klebsiella varicolla*

**Table 2:** Recent studies showing the dominant taxonomies in the group of women without GDM.

Study	Number of Women Surveyed GDM/Control (Total)	Gestational Age (Weeks)	Methods	Prevailing Taxonomies in the Group of Normoglycaemic Women
**Ye et al**. [53]	36/16 (52)	24–28	16s rRNA sequencing	*Faecalibacterium* spp.
**Cortez et al.** [43]	26/42 (68)	28–36	16s rRNA sequencing	*Bacterioides* spp.
**Crusell et al.** [39]	50/157 (207)	27–33	16s rRNA sequencing	*Marvinbryantia* spp., *Acetivibrio*, *Anaerosporobacter*
**Crusell et al.** [39]	43/81 (124)	21–29	Whole metagenome shotgun sequencing	*Bifidobacterium* spp., *Eubacterium* spp.

**Table 3:** Studies proving the epigenetic mechanisms influencing GDM.

**Ott et al.** [102]	Variable levels of DNA methylation in the promoter regions of the IR gene in SAT, VAT, CB and MB; Negative correlation between CpG4-GRE2, CpG3-AP-2 and SP1 methylation and the expression of the IR gene in SAT; Expression of the IR gene in CB positively related to methylation of the CpG2-AP-2 and SP1 region and negatively related to methylation of the CpG3-GRE1 region of the promoter of the IR gene.
**Haertle et al.** [105]	Lower level of methylation in the CpG2 region of the ATP5F1A gene in women with GDM;∙ Variable methylation levels of several CpG regions for MFAP4, PRKCH and HIF3A genes in women with GDM.
**Wu et al.** [101]	Variable methylation levels of CpG regions for COPS8, PIK3R5, HAAO, CCDC124, C5orf34 genes in women with GDM.
**Kelstrup et al.** [107]	Lower level of PPARGC1A gene expression in the muscle tissue of the offspring of women with GDM—a mechanism probably different than variable CpG methylation for the PPARGC1A gene;∙ Negative correlation between the expression of the PPARGC1A gene and the expression of the HOMA-IR gene in subcutaneous adipose tissue in children of mothers with GDM.
**Reichetzeder et al.** [98]	GDM strongly influences the level of placental DNA methylation in a way that is probably independent of other clinical factors.
**Gagne-Ouellet et al.** [108]	Decrease in the level of methylation in the CpG1 and CpG2 regions of the LPL gene in the placental tissues in patients with GDM;∙ Negative correlation between methylation at the LPL gene locus and its transcriptional activity.
**Hepp et al.** [109]	Decreased level of lysine acetylation in H3K9 in placental tissues during pregnancy complicated by GDM.
**Chang et al.** [110]	H3 and H4 deacetylation, H3K4 demethylation and H3K9 methylation negatively correlate with the expression of the PDX1 gene (IPF-1).
**Zhao et al.** [113]	GDM negatively correlates with the level of miRNA interacting with PAK1 on the beta cells of the pancreatic islets.
**Stirm et al.** [114]	Higher levels of miRNA-340 expression in peripheral blood lymphocytes in women with GDM.
**Balci et al.** [121]	Higher level of miRNA-7-5p expression in the blood serum of mothers with GDM.

## Data Availability

Not applicable.

## Abbreviations

The following abbreviations are used in this manuscript:

ADA	American Diabetes Association
BMI	Body Mass Index
EWAS	Epigenome-wide association studies
FFAs	Free fatty acids
GDM	Gestational diabetes mellitus
GM-CSF	Granulocyte-macrophage colony-stimulating factor
Hbd	Hebdomas
HOMA-IR	Homeostatic model assessment-insulin resistance
IEM	Inborn errors of metabolism
IGFBP1	Insulin like growth factor binding protein 1
Il	Interleukin
IPF1	Insulin promoter factor 1
LPL	Lipoprotein lipase
LPS	Lipopolysaccharides
MSRA	Methionine sulfoxde reductase A
PDX1	Pancreatic and duodenal homeobox 1
PPARGC1A	Peroxisome proliferator activated receptor gamma coactivator 1-alpha
spp.	species
TLR	Toll-like receptor
T2DM	Type II diabetes mellitus
